# Predicting need for intensive care unit admission in adult emphysematous pyelonephritis patients at emergency departments: comparison of five scoring systems

**DOI:** 10.1038/s41598-019-52989-7

**Published:** 2019-11-12

**Authors:** Xiao-Han Yap, Chip-Jin Ng, Kuang-Hung Hsu, Cheng-Yu Chien, Zhong Ning Leonard Goh, Chih-Huang Li, Yi-Ming Weng, Ming-Shun Hsieh, Hsien-Yi Chen, Joanna Chen-Yeen Seak, Chen-Ken Seak, Chen-June Seak

**Affiliations:** 1 College of Medicine, Chang Gung University, Taoyuan, Taiwan; 2 Department of Emergency Medicine, Lin-Kou Medical Center, Chang Gung Memorial Hospital, Taoyuan, Taiwan; 3 Laboratory for Epidemiology, Department of Health Care Management, and Healthy Aging Research Center, Chang Gung University, Taoyuan, Taiwan; 4 Department of Emergency Medicine, Ton-Yen General Hospital, Zhubei City, Hsinchu County Taiwan; 5 School of Medicine, International Medical University, Kuala Lumpur, Malaysia; 6 Department of Emergency Medicine, Prehospital Care Division, Taoyuan General Hospital, Ministry of Health and Welfare, Taoyuan, Taiwan; 7 Department of Emergency Medicine, Taipei Veterans General Hospital, Taoyuan Branch, Taoyuan, Taiwan; 8 School of Medicine, National Yang-Ming University, Taipei, Taiwan; 9 Institute of Occupational Medicine and Industrial Hygiene, College of Public Health, National Taiwan University, Taipei, Taiwan; 10 Sarawak General Hospital, Kuching, Sarawak, Malaysia

**Keywords:** Nephritis, Prognosis, Nephrology

## Abstract

This study assesses the performance of National Early Warning Score (NEWS), Quick Sepsis-related Organ Failure Assessment (qSOFA), Modified Early Warning Score (MEWS), Rapid Emergency Medicine Score (REMS), and Rapid Acute Physiology Score (RAPS) in predicting emphysematous pyelonephritis (EPN) patients’ need for intensive care unit (ICU) admission. A retrospective analysis was conducted at four training and research hospitals’ emergency departments (EDs) on all EPN adult patients from January 2007 to August 2017. Data extracted were used to calculate raw scores for five physiologic scoring systems. Mann-Whitney U tests and χ^2^ tests were done for numerical and categorical variables respectively to examine differences between characteristics of ICU and non-ICU patient populations. Predictability of ICU admission was evaluated with AUROC analysis. ICU patients had lower GCS scores, SpO2, platelet counts, and estimated glomerular filtration rate; and higher bands, blood urea nitrogen, creatinine, and incidences of septic shock and nephrectomy. NEWS performed best, with 73.85% accuracy at optimal cut-off of 3. In this multicentre ED EPN series, we recommend using NEWS in early identification of critical EPN patients and advance planning for ICU admission. This would reduce delays in ICU transfer and ultimately improve patient outcomes.

## Introduction

Emphysematous pyelonephritis (EPN) is defined as an acute, severe necrotising infection of the renal parenchyma and its surrounding tissues that results in the presence of gas in the renal parenchyma, collecting system, or perinephric tissue^1–5^. It has historically been associated with high mortality rates of up to 78% in the 1970s, due to poor recognition of this rare condition leading to delayed management of septic complications; early nephrectomy was the treatment of choice, though with the advent of advanced imaging computed tomography techniques, percutaneous drainage is now the favoured option with decreased mortality of 21%^6–9^. Together with early percutaneous drainage, comprehensive management of EPN comprise fluid resuscitation, aggressive antibiotic therapy, correction of reversible precipitating factors, and elective nephrectomy if still indicated after percutaneous drainage^10–12^.

Despite the critical nature of these EPN patients, there is no global consensus of management algorithms for them in the emergency department (ED). Close, round-the-clock monitoring in the intensive care unit (ICU) is therefore warranted, so that clinicians can detect failure of conservative treatment promptly and perform timely percutaneous drainage and/or emergency nephrectomy.

Objective criteria to assess and predict an EPN patient’s need for ICU admission has however yet to be established despite its importance – admitting a patient who does not require ICU care exacerbates the overcrowding situation often seen in the ICU; on the contrary, failure or delays in admitting a patient who requires ICU care is associated with higher mortality rates^13^, potentially worsening survival further in this EPN patient population.

General guidelines for admission to the ICU are available from the Society of Critical Care Medicine^14^, though these recommendations are highly dependent on clinical expertise and experience. Additionally, there may be differences in opinions among emergency physicians (EPs), surgeons, and intensivists on whether ICU admission is really necessary.

National Early Warning Score (NEWS)^15^, Quick Sepsis Related Organ Failure Assessment (qSOFA)^16^, Modified Early Warning Score (MEWS)^17^, Rapid Emergency Medicine Score (REMS)^18^, and Rapid Acute Physiology Score (RAPS)^19^ (see Supplementary Tables S1–S5 online) are five physiologic scoring systems commonly used in the ED. These scoring systems consist of various readily-available parameters, thus allowing for point-of-care use to objectively assess the severity of an ED patient’s clinical condition and predict patient mortality.

We previously studied the use of such scoring systems in predicting mortality of patients with hepatic venous portal gas, splenic abscess, and renal abscess^20–23^. We found that Mortality in Emergency Department Sepsis score (MEDS) was superior to MEWS, REMS, and RAPS due to its incorporation of patient characteristics. We however excluded this score for the purposes of this study, as its basis of superiority in predicting mortality did not apply to the forecasting of need for ICU admission. In particular, the variable of “terminal illness” had the highest weightage in MEDS, yet such patients are generally managed palliatively in the general ward rather than aggressively in the ICU. With the utility of MEDS in predicting ICU admission curtailed, we substituted it with two other commonly used scoring systems, NEWS and qSOFA, in the design of this study.

This study examines the use of the aforementioned scoring systems to predict the need for ICU admission in EPN patients presenting to the ED. These results will assist EPs, surgeons, and intensivists in early identification of such critically-ill patients, enabling them to make the necessary arrangements with the ICU staff ahead of time.

## Materials and Methods

### Study design

A retrospective analysis was conducted at the EDs of four training and research hospitals of the respective sizes: Linkou Chang Gung Memorial Hospital (3406 beds, 17000 monthly ED visits); Kaohsiung Chang Gung Memorial Hospital (2686 beds, 12000 monthly ED visits); Chiayi Chang Gung Memorial Hospital (1375 beds, 5800 monthly ED visits); and Keelung Chang Gung Memorial Hospital (1089 beds, 5700 monthly ED visits). The Chang Gung Medical Foundation Institutional Review Board approved this study for all four hospitals (IRB No. 201701502B0C501), waiving the need for consent from study participants. Data was accessed anonymously. All methods were carried out in accordance with the relevant guidelines and regulations.

### Settings and subjects

Patients older than 18 years admitted to the EDs of the four hospitals with EPN (diagnosed via clinical presentation and contrast-enhanced abdominal computed tomography scan) from January 2007 to August 2017 were recruited.

### Measurement of variables

The following information were extracted from their medical records: age, sex, clinical presentation, temperature, heart rate, respiratory rate, blood pressure, Glasgow Coma Scale (GCS) score, radiographic imaging, and any other relevant data. The worst values observed during the patients’ ED stays were recorded. NEWS, qSOFA, MEWS, REMS, and RAPS were subsequently calculated for each patient. The study endpoint was admission to the ICU ward.

### Criteria for ICU admission

Due to lack of consensus on specific international guidelines in determining need for ICU admission, our ICU committee has decided on the below-mentioned criteria to maximise patient prognoses and outcomes while reducing ICU overcrowding. These criteria have remained consistent throughout the study period.

The general criteria for ICU admission in our hospitals include at least one of the following: (1) requiring or likely to require advanced respiratory support; (2) requiring support of two or more organ systems; and (3) had chronic impairment of one or more organ systems and currently require support for an acute reversible failure of another organ.

### Statistical analysis

Descriptive statistics were presented as median with interquartiles for numerical variables and frequencies with percentages (%) for categorical variables. Mann-Whitney U tests and χ^2^ tests were done for numerical and categorical variables respectively to examine the differences between characteristics of ICU and non-ICU patient populations^24^. Receiver operating characteristic curves were plotted for each score using logistic regression, to obtain the area under curve (AUC) value for comparison of ICU admission predictability. Optimal cut-off points were subsequently identified using Youden’s index, and the corresponding sensitivity, specificity, and accuracy rates calculated. DeLong test was also used to compare the AUC of NEWS with that of other scores. Statistical significance was taken at p < 0.05.

## Results

A total of 65 patients aged between 33 and 89 years were identified in the four hospitals over a span of 10 years and 8 months. All recruited patients underwent abdominal CT scans. All patients were admitted to ICUs or general wards. None of them died in the EDs prior to admissions and none were transferred to other hospitals. Compared to the group of EPN patients who did not require ICU admission, those who were admitted to the ICU had lower GCS scores (p = 0.0070), lower SpO_2_ (p = 0.0036), higher incidence of septic shock (p < 0.0001), lower platelet counts (p = 0.0204), bandemia (p = 0.0058), higher blood urea nitrogen (p = 0.0123), higher creatinine (p = 0.0009), lower estimated glomerular filtration rate (p = 0.0010), higher incidence of nephrectomy (p < 0.0001) (Table 1).

**Table 1:** Characteristics of non-ICU versus ICU patients.

	Patients		
	Non-ICU	ICU	p-value
**No**.	44	21	
**Age, Median (IQR)**	57 (51–69)	64 (56–76)	0.1872
**Sex, No. (%)**			0.9900
Male	10 (22.73)	4 (19.05)	
Female	34 (77.27)	17 (80.95)	
**Major Predisposing factors, No. (%)**			
Diabetes mellitus	38 (86.36)	19 (90.48)	0.9900
Urinary obstruction	14 (31.82)	7 (33.33)	0.9028
Renal failure	13 (29.55)	8 (38.1)	0.4906
**Vital sign, Median (IQR)**			
Body Temperature (°C)	37.1 (36.4–37.9)	37.3 (36.4–38.2)	0.7372
Pulse rate (/min)	100.5 (91.5–113)	111 (98–120)	0.1759
Respiratory rate (/min)	19.5 (18–20)	20 (18–22)	0.7062
SpO_2_ (%)*	97.4 (98–96)	88 (97–87)	0.0036
Glasgow Coma Scale, No. (%)*			0.0070
≤8	1 (2.27)	3 (14.29)	
9~11	1 (2.27)	4 (19.05)	
≥12	42 (95.45)	14 (66.67)	
**Clinical presentations, No. (%)**			
Fever	29 (65.91)	12 (57.14)	0.4934
Dysuria / Pyuria	23 (52.27)	8 (38.1)	0.2845
Nausea / Vomiting	12 (27.27)	4 (19.05)	0.4716
Flank pain	26 (59.09)	8 (38.1)	0.1130
Abdominal pain	10 (22.73)	9 (42.86)	0.0952
Septic shock*	2 (4.55)	10 (47.62)	<0.0001
**Laboratory results, Median (IQR)**			
Leukocyte count (×10^3^/μL)	13.45 (9.6–18.1)	14.5 (7.6–18.7)	0.9833
Haematocrit (%)	31.85 (27.2–35.05)	30.7 (25.6–33.9)	0.5255
Platelets (×10^3^/μL)*	192.5 (118.5–289)	105 (64–221)	0.0204
Segment (%)	83.5 (75.75–90.85)	82 (76–88)	0.4389
Band (%)*	0 (0–2)	3 (0.5–8)	0.0058
Blood glucose (mg/dL)	287 (144.5–489.5)	270 (183–515)	0.8917
Blood Urea Nitrogen (mg/dL)*	25 (10–48)	46.6 (35–65)	0.0123
Creatinine (mg/dL)*	1.135 (0.79–2.49)	2.6 (1.8–4.04)	0.0009
Estimated Glomerular Filtration Rate (ml/min/1.73 m^2^)*	44.55 (22.32–82.22)	18.25 (12.49–26.62)	0.0010
**Involved Kidney, No. (%)**			0.9935
Left	23 (52.27)	11 (52.38)	
Right	21 (47.73)	10 (47.62)	
**Management, No. (%)**			
Antibiotics without invasive procedure	8 (18.18)	1 (4.76)	0.2512
Percutaneous drainage	29 (65.91)	9 (42.86)	0.0778
Surgical drainage	6 (13.64)	3 (14.29)	0.9900
Nephrectomy*	1 (2.27)	9 (42.86)	<0.0001
Symptom onset to ED presentation (days), Median (IQR)*	4 (2–7)	1 (1–3)	0.0095
ED presentation to ward/ICU admission (hrs), Median (IQR)	14.9 (5.6–25.9)	11 (3.8–20.8)	0.3333
**ED presentation to operation (hrs)**,			
Median (IQR)	21 (2.5–120)	10 (5–46)	0.3641
Days of admission to general ward, Median(IQR)	20.5 (13.5–31.5)	16 (10–33)	0.0760
Days of admission to ICU, Median(IQR)*	0 (0–0)	5 (4–8)	<0.0001
Mortality, No. (%)*	0 (0)	9 (42.86)	<0.0001

* indicates a statistically significant difference between non-ICU and ICU patients.

**Figure 1:**
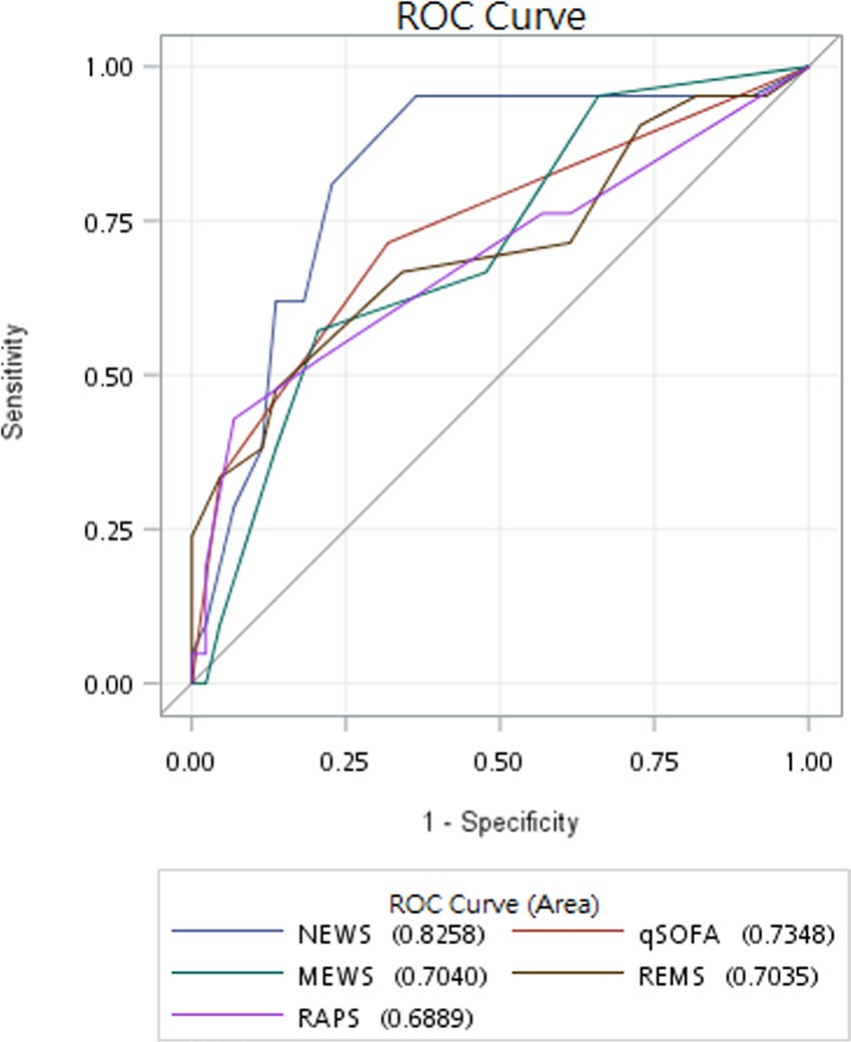
Receiver operating curves for predicting ICU admission according to the NEWS, qSOFA, MEWS, REMS, and RAPS scoring systems.

AUROC analysis demonstrated the predictability of the five scoring systems as such, listed in descending order: NEWS, 0.8258; qSOFA, 0.7348; MEWS, 0.7040; REMS, 0.7035; RAPS, 0.6889 (Fig. 1). NEWS was found to be the best in predicting the need for ICU admission, with an optimal cut-off point of 3, sensitivity of 95.24%, specificity of 63.64%, positive predictive value of 55.56%, and negative predictive value of 96.55%. That of the other four physiologic scoring systems can be found in Table 2. Youden’s indices for the respective scores were as follows: NEWS 0.5887; qSOFA 0.3961; MEWS 0.3669; REMS 0.3398; and RAPS 0.3604. DeLong test found AUC of NEWS to be significantly larger than that of MEWS (p = 0.0256) and RAPS (p = 0.0486) (Table 3).

**Table 2:** Accuracies, sensitivities, specificities, and predictive values for NEWS, qSOFA, MEWS, REMS, and RAPS in predicting ICU admission.

	Non-ICU Median (IQR)	ICU Median (IQR)	Optimal cut-off	Accuracy	Sen	Sp	PPV	NPV	False + ve	False −ve
NEWS	2 (1,3)	6 (4,8)	3	73.85%	95.24%	63.64%	55.56%	96.55%	24.73%	1.52%
qSOFA	0 (0,1)	1 (0,2)	1	69.23%	71.43%	68.18%	51.72%	83.33%	21.64%	9.14%
MEWS	2 (1,3)	4 (2,5)	4	72.31%	57.14%	79.55%	57.14%	79.55%	13.91%	13.71%
REMS	5 (3,6)	6 (4,9)	7	73.85%	47.62%	86.36%	62.50%	77.55%	9.27%	16.76%
RAPS	2 (0,2)	3 (2,4)	4	76.92%	42.86%	93.18%	75.00%	77.36%	4.64%	18.29%

**Table 3:** DeLong analyses comparing AUC of NEWS with that of qSOFA, MEWS, REMS, and RAPS in predicting ICU admission.

Comparators	Difference between AUCs	95% CI	p-value
NEWS vs qSOFA	0.0909	(−0.0193, 0.2010)	0.1060
NEWS vs MEWS	0.1220	(0.0149, 0.2290)	0.0256
NEWS vs REMS	0.1220	(−0.0495, 0.2940)	0.1630
NEWS vs RAPS	0.1370	(0.0009, 0.2730)	0.0486

## Discussion

This is a multi-centre study into EPN patients at the ED, as well as the first to assess the suitability of ED scoring systems in predicting ICU admission requirements for these patients. Five scoring systems were compared, and NEWS was found to be the best performer.

NEWS is an early warning score devised by the National Early Warning Score Development and Implementation Group (NEWSDIG) for the Royal College of Physicians to identify acutely ill patients requiring urgent assessment by a critical care team to determine necessity of escalation of care. It has been shown to be versatile, and has been validated for use in acute medical admissions^15^, non-elective surgical admissions^25^, and even in pre-hospital settings^26,27^. Previous studies have also found NEWS to be equal or superior to other commonly-used risk stratification tools, such as qSOFA, Systemic Inflammatory Response Syndrome score, and the medical emergency team criteria^28,29^.

Patients are classified into three categories dependent on their raw scores. Evaluation by a competent registered nurse is sufficient for a patient with a low score (NEWS 1–4), while those with a medium score (NEWS 5–6) should be reviewed by a ward-based doctor or acute team nurse. Patients with high scores (NEWS ≥7) require prompt emergency assessment by a clinical team skilled in critical care, and they are usually transferred to a higher dependency care area^15^.

Our similar study findings thus support the original recommendations of NEWS. EPN patients with a NEWS of the cut-off point 3 and below were unlikely to require ICU admission (NPV 96.55%) and can generally be transferred to the general ward. On the other hand, it is preferable to plan for ICU admission of those with scores of 4 and above, since the clinical situation of EPN patients can deteriorate rapidly without warning.

The overall mortality rate of patients admitted to the ICU in our study was 42.86%. We re-evaluated these cases and found that all 9 patients indeed had a NEWS above the optimal cut-off of 3, and thus would have been promptly identified. Furthermore, the low false negative incidence of 1.52% supports the conclusion that NEWS is a good discriminator in identifying EPN patients requiring ICU admission. This would ultimately streamline the decision-making process and improve quality of patient care.

Two other well-performing scores include qSOFA and MEWS, with cut-off scores in the studied EPN population which reflect the original designed intentions of each respective score. On the contrary, RAPS performed poorly, most probably since it was devised for use in a non-surgical ED patient population. This suggests that it is crucial for choice of any scoring system to take into consideration whether the patient in question is similar to the population for which the score was originally validated.

Further analysis with DeLong test confirmed the superiority of NEWS over MEWS and RAPS. DeLong test also favoured NEWS over qSOFA and REMS; with an increasing population size, the results should prove significant.

Upon examination of differences in characteristics between ICU and non-ICU patients, the statistically significant ones are as expected in a severely ill EPN patient. ICU patients were more likely to be in septic shock (p < 0.0001) with corresponding oxygen desaturation (p = 0.0036), lower GCS scores (p = 0.0070), lower platelet count (p = 0.0204), and bandemia (p = 0.0058) as well as worse renal function (higher BUN, p = 0.0123; higher creatinine, p = 0.0009; lower eGFR, p = 0.0010). Consequently, more ICU patients required nephrectomy (p < 0.0001). This then manifested as derangements in the EPN patients’ vital signs, which were incorporated in the NEWS physiologic scoring system.

NEWS is therefore an ideal objective assessment tool for the evaluation of need for ICU admission in EPN patients presenting to the ED. Its simplicity means that junior healthcare staff, including ED nurses, can employ the score even without much prior training, reducing uncertainty in patient management plans when senior physicians are tending to more urgent emergencies. Beds in the ICU can then be reserved for identified EPN patients to allow for seamless transfer. This pre-planned resource allocation and prioritization based on NEWS helps improve care efficiency and ultimately patient outcomes.

While results of this study is confined to EPN patients, further research can be done into the application of NEWS to other intra-abdominal infections. Furthermore, although this is a sizeable multicentre study comparatively to date into the population of EPN patients in the ED, the sample size is still small. Larger prospective studies are required to validate the results.

Looking into further improvements of the current NEWS system may also be warranted in the future. Particularly, the incidence of false positives and false negatives can be reduced by inclusion of supplementary indicators when conducting similar studies with larger sample populations.

## Conclusion

In this multicentre ED EPN series, NEWS is the best performing physiologic score among the five scoring systems studied in identifying EPN patients who require ICU admission. Its simplicity allows for junior healthcare staff, including nurses, to utilize it for patient assessment without much prior training. We recommend using it in the planning of management of EPN patients, so as to reduce delays in transfer to the ICU and ultimately improve patient outcomes.

## Supplementary information

NEWS, qSOFA, MEWS, REMS and RAPS

## Data Availability

The datasets generated during and/or analysed during the current study are available from the corresponding author on reasonable request.

## References

[1] Michaeli J, Mogle S, Perlberg S, Heiman S, Caine M (1984). Emphysematous pyelonephritis. J. Urol..

[2] Pontin AR, Barnes RD, Joffe J, Kahn D (1995). Emphysematous pyelonephritis in diabetic patients. Br. J. Urol..

[3] Ubee SS, McGlynn L, Fordham M (2011). Emphysematous pyelonephritis. BJU Int..

[4] Kumar A, Turney JH, Brownjohn AM, McMahon MJ (2001). Unusual bacterial infections of the urinary tract in diabetic patients–rare but frequently lethal. Nephrol. Dial. Transplant..

[5] Evanoff GV, Thompson CS, Foley R, Weinman EJ (1987). Spectrum of gas within the kidney. Emphysematous pyelonephritis and emphysematous pyelitis. Am. J. Med..

[6] Shokeir AA, El-Azab M, Mohsen T, El-Diasty T (1997). Emphysematous pyelonephritis: a 15-year experience with 20 cases. Urology..

[7] Ahlering TE (1985). Emphysematous pyelonephritis: a 5-year experience with 13 patients. J. Urol..

[8] Wan YL, Lee TY, Bullard MJ, Tsai CC (1996). Acute gas-producing bacterial renal infection: correlation between imaging findings and clinical outcome. Radiology..

[9] Chen MT (1997). Percutaneous drainage in the treatment of emphysematous pyelonephritis: 10-year experience. J. Urol..

[10] Huang JJ, Tseng CC (2000). Emphysematous pyelonephritis: clinicoradiological classification, management, prognosis, and pathogenesis. Arch. Intern. Med..

[11] Somani BK (2008). Is percutaneous drainage the new gold standard in the management of emphysematous pyelonephritis? Evidence from a systematic review. J. Urol..

[12] Kapoor R (2010). Predictive factors for mortality and need for nephrectomy in patients with emphysematous pyelonephritis. BJU Int..

[13] Yu BH (2014). Delayed admission to intensive care unit for critically surgical patients is associated with increased mortality. Am. J. Surg..

[14] Nates JL (2016). ICU admission, discharge, and triage guidelines: a framework to enhance clinical operations, development of institutional policies, and further research. Crit. Care. Med..

[15] Smith GB, Prytherch DR, Meredith P, Schmidt PE, Featherstone PI (2013). The ability of the National Early Warning Score (NEWS) to discriminate patients at risk of early cardiac arrest, unanticipated intensive care unit admission, and death. Resuscitation..

[16] Seymour CW (2016). Assessment of clinical criteria for sepsis: for the Third International Consensus Definitions for Sepsis and Septic Shock (Sepsis-3). JAMA..

[17] Subbe CP, Kruger M, Rutherford P, Gemmel L (2001). Validation of a modified Early Warning Score in medical admissions. QJM..

[18] Olsson T, Terent A, Lind L (2004). Rapid Emergency Medicine score: a new prognostic tool for in-hospital mortality in nonsurgical emergency department patients. J. Intern. Med..

[19] Rhee KJ, Fisher CJ, Willitis NH (1987). The Rapid Acute Physiology Score. Am. J. Emerg. Med..

[20] Seak CJ (2014). Performance assessment of the Simplified Acute Physiology Score II, the Acute Physiology and Chronic Health Evaluation II score, and the Sequential Organ Failure Assessment score in predicting the outcomes of adult patients with hepatic portal venous gas in the ED. Am. J. Emerg. Med..

[21] Seak CJ (2017). Rapid Emergency Medicine Score: a novel prognostic tool for predicting the outcomes of adult patients with hepatic portal venous gas in the emergency department. PLoS ONE..

[22] Hung SK (2017). Comparison of the Mortality in Emergency Department Sepsis Score, Modified Early Warning Score, Rapid Emergency Medicine Score, and Rapid Acute Physiology Score for predicting the outcomes of adult splenic abscess patients in the emergency department. PLoS ONE..

[23] Chang SH (2018). Performance assessment of the Mortality in Emergency Department Sepsis Score, modified Early Warning Score, Rapid Emergency Medicine Score, and Rapid Acute Physiology Score in predicting survival outcomes of adult renal abscess patients in the emergency department. Biomed. Res. Int..

[24] Zhang Z (2016). Univariate description and bivariate statistical inference: the first step delving into data. Ann Transl Med..

[25] Kovacs C (2016). Comparison of the National Early Warning Score in non-elective medical and surgical patients. Br. J. Surg..

[26] Silcock DJ, Corfield AR, Gowens PA, Rooney KD (2015). Validation of the National Early Warning Score in the prehospital setting. Resuscitation..

[27] Shaw J, Fothergill RT, Clark S, Moore F (2017). Can the prehospital National Early Warning Score identify patients most at risk from subsequent deterioration?. Emerg. Med. J..

[28] Goulden R (2018). qSOFA, SIRS and NEWS for predicting inhospital mortality and ICU admission in emergency admissions treated as sepsis. Emerg. Med. J..

[29] Smith GB (2016). A comparison of the ability of the physiologic components of medical emergency team criteria and the U.K. National Early Warning Score to discriminate patients at risk of a range of adverse clinical outcomes. Crit. Care. Med..

