# Supplementary material for: Predicting need for intensive care unit admission in adult emphysematous pyelonephritis patients at emergency departments: comparison of five scoring systems
Source: Sci Rep. 2019 Nov 12;9:16618. doi: 10.1038/s41598-019-52989-7 (PMC6851397; doi:10.1038/s41598-019-52989-7)
Supplement: Supplementary file 1 — NEWS, qSOFA, MEWS, REMS and RAPS [file 41598_2019_52989_MOESM1_ESM.docx]

**Predicting need for intensive care unit admission in adult emphysematous pyelonephritis patients at emergency departments: comparison of five scoring systems**

Xiao-Han Yap^1,2^; ^¶^Chip-Jin Ng, MD^1,2^; Kuang-Hung Hsu, PHD^2,3^; Cheng-Yu Chien, MD^4^; Zhong Ning Leonard Goh, MBBS^5^; Chih-Huang Li, MD, PHD^1,2^; Yi-Ming Weng, MD^6^; Ming-Shun Hsieh, MD^7,8,9^; Hsien-Yi Chen, MD^1,2^; Joanna Chen-Yeen Seak, MD^10^; Chen-Ken Seak, MBBS^10^; *Chen-June Seak, MD^1,2,9^

^1^ College of Medicine, Chang Gung University, Taoyuan, Taiwan

^2^ Department of Emergency Medicine, Lin-Kou Medical Center, Chang Gung Memorial Hospital, Taoyuan, Taiwan

^3^ Laboratory for Epidemiology, Department of Health Care Management, and Healthy Aging Research Center, Chang Gung University, Taoyuan, Taiwan

^4^ Department of Emergency Medicine, Ton-Yen General Hospital, Zhubei City, Hsinchu County, Taiwan

^5^ School of Medicine, International Medical University, Kuala Lumpur, Malaysia

^6^ Department of Emergency Medicine, Prehospital Care Division, Taoyuan General Hospital, Ministry of Health and Welfare, Taoyuan, Taiwan

^7^ Department of Emergency Medicine, Taipei Veterans General Hospital, Taoyuan Branch, Taoyuan, Taiwan

^8^ School of Medicine, National Yang-Ming University, Taipei, Taiwan

^9^ Institute of Occupational Medicine and Industrial Hygiene, College of Public Health, National Taiwan University, Taipei, Taiwan

^10^ Sarawak General Hospital, Kuching, Sarawak, Malaysia

^¶^ **Xiao-Han Yap** and **Chip-Jin Ng** are the first authors. The first two authors contributed equally to this article.

**Supplementary Table 1. National Early Warning Score (NEWS)**

| Score | | | | |
| --- | --- | --- | --- | --- |
| Variable | 0 | +1 | +2 | +3 |
| Respiratory rate (/min) | 12–20 | 9–11 | 21–24 | ≤8  ≥25 |
| SpO_2_ (%) | ≥96 | 94–95 | 92–93 | ≤91 |
| Supplemental oxygen? | No | Yes |  |  |
| Temperature (℃) | 36.1–38.0 | 35.1–36.0  38.1–39.0 | ≥39.1 | ≤35 |
| Systolic BP (mmHg) | 111–219 | 101–110 | 91–100 | ≤90  ≥220 |
| Heart rate (/min) | 51–90 | 41–50  91–110 | 111–130 | ≤40  ≥131 |
| AVPU score | **A**lert |  |  | Reacts to **V**oice  Reacts to **P**ain  **U**nresponsive |

SpO_2_, peripheral oxygen saturation; BP, blood pressure

**Supplementary Table 2. Quick Sepsis-related Organ Failure Assessment (qSOFA)**

| Score | | |
| --- | --- | --- |
| Variable | Yes | No |
| Altered mental status, GCS < 15 | +1 | 0 |
| Respiratory rate ≥ 22/min | +1 | 0 |
| Systolic blood pressure ≤100 mmHg | +1 | 0 |

**Supplementary Table 3. Modified Early Warning Score (MEWS)**

| Score | | | | |
| --- | --- | --- | --- | --- |
| Variable | 0 | +1 | +2 | +3 |
| Systolic BP (mmHg) | 101–199 | 81–100 | 71–80  ≥200 | <70 |
| Heart rate (/min) | 51–100 | 41–50  101–110 | <40  111–129 | ≥130 |
| Respiratory rate (/min) | 9–14 | 15–20 | <9  21–29 | ≥30 |
| Temperature (℃) | 35–38.4 |  | <35  ≥38.5 |  |
| AVPU score | **A**lert | Reacts to **V**oice | Reacts to **P**ain | **U**nresponsive |

**Supplementary Table 4. Rapid Emergency Medicine Score (REMS)**

|  | Score |  |  |  |  |  |  |
| --- | --- | --- | --- | --- | --- | --- | --- |
| Variable | 0 | +1 | +2 | +3 | +4 | +5 | +6 |
| Age (years) | <45 |  | 45–54 | 55–64 |  | 65–74 | >74 |
| PR (/min) | 70–109 |  | 55–69  110–139 | 40–54  140–179 | ≤39  >179 |  |  |
| MAP (mmHg) | 70–109 |  | 50–69  110–129 | 130–159 | ≤49  >159 |  |  |
| RR (/min) | 12–24 | 10–11  25–34 | 6–9 | 35–49 | ≤5  >49 |  |  |
| GCS | 14 or 15 | 11–13 | 8–10 | 5–7 | 3 or 4 |  |  |
| SpO_2_ (%) | >89 | 86–89 |  | 75–85 | <75 |  |  |

PR, pulse rate; MAP, mean arterial pressure; RR, respiratory rate; GCS, Glasgow Coma Scale; SpO_2_, peripheral oxygen saturation

**Supplementary Table 5. Rapid Acute Physiology Score (RAPS)**

|  | Score |  |  |  |  |
| --- | --- | --- | --- | --- | --- |
| Variable | 0 | +1 | +2 | +3 | +4 |
| PR (/min) | 70–109 |  | 55–69  110–139 | 40–54  140–179 | ≤39  ≥180 |
| MAP (mmHg) | 70–109 |  | 50–69  110–129 | 130–159 | ≤49  ≥160 |
| RR (/min) | 12–24 | 10–11  25–34 | 6–9 | 35–49 | ≤5  ≥50 |
| GCS | ≥14 | 11–13 | 8–10 | 5–7 | ≤4 |

PR, pulse rate; MAP, mean arterial pressure; RR, respiratory rate; GCS, Glasgow Coma Scale
